# RIAM-VASP Module Relays Integrin Complement Receptors in Outside-In Signaling Driving Particle Engulfment

**DOI:** 10.3390/cells9051166

**Published:** 2020-05-08

**Authors:** Alvaro Torres-Gomez, Jose Luis Sanchez-Trincado, Víctor Toribio, Raul Torres-Ruiz, Sandra Rodríguez-Perales, María Yáñez-Mó, Pedro A. Reche, Carlos Cabañas, Esther M. Lafuente

**Affiliations:** 1 Department of Immunology, Ophthalmology and Otorhinolaryngology, School of Medicine, Universidad Complutense de Madrid, and Instituto (I+12), 28040 Madrid, Spain; atorr01@ucm.es (A.T.-G.); josels07@ucm.es (J.L.S.-T.); parecheg@med.ucm.es (P.A.R.); ccabanas@cbm.csic.es (C.C.); 2 Severo Ochoa Center for Molecular Biology (CSIC-UAM), 28049 Madrid, Spain; viktoribio88@hotmail.com (V.T.); maria.yannez@uam.es (M.Y.-M.); 3 Departamento de Biología Molecular, Universidad Autónoma de Madrid (UAM) and Instituto de Investigación Sanitaria La Princesa (IIS-IP), 28049 Madrid, Spain; 4 Molecular Cytogenetics and Genome Editing Unit, Human Cancer Genetics Program, Centro Nacional de Investigaciones Oncológicas (CNIO), 28029 Madrid, Spain; rtorresr@cnio.es (R.T.-R.); srodriguezp@cnio.es (S.R.-P.); 5 Josep Carreras Leukemia Research Institute and Department of Biomedicine, School of Medicine, University of Barcelona, 08007 Barcelona, Spain

**Keywords:** phagocytosis, complement, CR3, CR4, Mac-1, β_2_ integrins, RIAM, VASP, outside-in

## Abstract

The phagocytic integrins and complement receptors α_M_β_2_/CR3 and α_X_β_2_/CR4 are classically associated with the phagocytosis of iC3b-opsonized particles. The activation of this receptor is dependent on signals derived from other receptors (inside-out signaling) with the crucial involvement of the Rap1-RIAM-Talin-1 pathway. Here, we analyze the implication of RIAM and its binding partner VASP in the signaling events occurring downstream of β_2_ integrins (outside-in) during complement-mediated phagocytosis. To this end, we used HL-60 promyelocytic cell lines deficient in RIAM or VASP or overexpressing EGFP-tagged VASP to determine VASP dynamics at phagocytic cups. Our results indicate that RIAM-deficient HL-60 cells presented impaired particle internalization and altered integrin downstream signaling during complement-dependent phagocytosis. Similarly, VASP deficiency completely blocked phagocytosis, while VASP overexpression increased the random movement of phagocytic particles at the cell surface, with reduced internalization. Moreover, the recruitment of VASP to particle contact sites, amount of pSer157-VASP and formation of actin-rich phagocytic cups were dependent on RIAM expression. Our results suggested that RIAM worked as a relay for integrin complement receptors in outside-in signaling, coordinating integrin activation and cytoskeletal rearrangements via its interaction with VASP.

## 1. Introduction

Essential immunological processes like immune synapse formation, cell migration, leukocyte extravasation, neutrophil extracellular trap formation, NK cell killing and phagocytosis depend on integrin activation and the recognition of their ligands [1,2,3,4]. Of special note are the integrins α_M_β_2_ and α_X_β_2_ (also known as CR3, CD11b/CD18, Mac-1 and CR4, CD11c/CD18, respectively), which are classically associated with the phagocytosis of iC3b-opsonized particles, cellular debris and pathogens [5].

In resting cells, these integrins remain inactive in a low affinity conformation. Activation can be induced by signals stemming from different receptors (GPCR, FcγR, CD44, TLRs, etc) upon the recognition of their ligands such as inflammatory moieties (e.g., LPS or fMLP) or cytokines (e.g., TNFα) through an inside-out signaling process [6,7,8,9]. Receptor downstream signaling induces Talin recruitment to β_2_ integrin tails, α and β subunit tail separation, and the extension of the ectodomain, promoting the active conformation with a high affinity for ligands [10,11,12]. Alternatively, ligand binding, extracellular Mn^2+^, and mechanical forces acting outside the cell can induce integrin conformational changes supporting tail separation, intracellular protein recruitment and signaling cascades known as outside-in signaling [13,14]. Outside-in and inside-out signaling processes may involve different intracellular partners and/or protein interactions, yet Talin recruitment to α_M_β_2_ may be important for both pathways [15].

Talin recruitment is a limiting step in integrin activation, and RIAM (Rap1-Interacting Adaptor Molecule) emerges as a key molecule in phagocytosis. RIAM interacts with membrane-bound Rap1-GTP through its central Ras association domain [16], being recruited to the plasma membrane where it recognizes phosphatidylinositides via its pleckstrin homology domain [17,18]. RIAM interacts with Talin through its N-terminal region [19,20], and this interaction releases Talin from its basal auto-inhibited state, allowing its interaction with β_2_ integrin tails, which brings about integrin activation [21]. Additionally, RIAM interacts with PLCγ1, Profilin and VASP via proline-rich regions [16,22,23].

RIAM has a critical role in α_M_β_2_ activation during complement-mediated phagocytosis. RIAM knockdown in promyelocytic cell lines and monocyte-derived macrophages reduces phagocytosis in response to LPS and fMLP, diminishes the expression of the activation-reporter epitope CBRM1/5 and impairs Talin recruitment to β_2_ integrins [24]. RIAM-null polymorphonuclear leukocytes display a reduced uptake of serum-opsonized bacteria and diminished free reactive oxygen species production, pointing to a role of RIAM in β_2_ integrin-mediated outside-in signaling [25].

Outside-in signaling promotes the actin cytoskeleton remodeling required for particle engulfment. Vasodilator-stimulated phosphoprotein (VASP) emerges as potential candidate involved in these changes [26]. VASP localizes at dynamic structures, such as filopodia and lamellipodia, and has a processive actin polymerase activity, producing long linear actin filaments and subsequent membrane protrusions [27].

VASP function is differentially modulated by phosphorylation at several residues [28]. In vitro actin polymerization is blocked by phosphorylation at Ser^239^ and Thr^278^, whilst that at pSer^157^ has no impact on that process but dictates VASP membrane localization [29,30]. In cellular systems, however, pSer^157^ promotes membrane ruffling, suggesting actin polymerization [28,31,32].

Taking into account these precedents, we hypothesized that during complement-mediated phagocytosis, RIAM may link integrin activation to the actin cytoskeleton via outside-in signaling involving VASP. Using promyelocytic RIAM and VASP deficient HL-60 cells, we found that RIAM is required for particle internalization after outside-in stimulation. The importance of VASP in this process was highlighted by the fact that VASP overexpression increased the random movement of phagocytic particles at the cell surface with reduced internalization, whilst the abrogation of VASP expression completely blocked engulfment. Finally, we demonstrate that RIAM expression determined VASP localization at the phagocytic cup and its level of phosphorylation at Ser^157^. These results point to RIAM as a key organizer of the whole phagocytic process, relaying integrin conformational changes to F-actin through VASP.

## 2. Materials and Methods

### 2.1. Cell Cultures

Human promyelocytic HL-60 (ATCC: CCL-240) and derived cell lines were cultured in 10 mL of RPMI 1640 medium with 10% (*v*/*v*) fetal-calf serum (FCS), 1% (*v*/*v*) glutamine and 1% (*w*/*v*) penicillin-streptomycin (Lonza, Basel, Switzerland) [33] using Nunc™ 100 mm dishes (Thermo Scientific, Waltham, MA, USA). The cells were passed every two days and seeded at a cell density of 2.5 × 10^5^ cells/mL The cells were differentiated into neutrophil-like HL-60 using 1 μM retinoic acid (Sigma, St. Louis, MO, USA), city, state abbr. if USA, country) for at least 2 days. The cells were routinely tested for mycoplasma contamination, and only mycoplasma-negative cells were used in this study.

### 2.2. Phagocytosis Assays

Phagocytosis assays were carried out as previously described [24]. Fresh sheep red blood cells (RBCs) (Thermo Scientific, Waltham, MA, USA) were labelled with 2 μM DDAO (Invitrogen, Waltham, MA, USA) at 37 °C for 20 min. Excess DDAO was removed by washing with RPMI 10% FCS, reserving an aliquot of labeled unopsonized RBCs. Opsonization was carried out by incubation with sub-agglutinating concentrations of polyclonal rabbit IgM anti-sheep RBC cells (MyBioSource, San Diego, CA, USA) and later treatment with 10% C5-deficient human serum (Sigma, St. Louis, MO, USA) for complement opsonization. Differentiated HL-60 cells were harvested and starved for 3 h in serum-free RPMI, subsequently treated with either 320 nM LPS (Sigma) or 1 mM MnCl_2_, and incubated for 30 or 5 min, respectively. The cells were then incubated in a 1:10 ratio with complement-opsonized RBCs (C3-RBC) or unopsonized RBCs as a negative control, for 30 min at 37 °C, and unbound RBCs were removed by washing thrice with ice-cold PBS. To determine particle internalization, cell-bound RBCs were exposed to a 30 s hypotonic shock with distilled H_2_O and isotonicity restored through the addition of an equal volume of twice-concentrated PBS.

Cells were analyzed using a BD FACSCalibur II flow cytometer (BD Biosciences, San Diego, CA, USA) and the data were analyzed using the FlowJo package (BD Biosciences) and expressed as Association Index (AI), indicating the amount of cells with attached and engulfed particles, or Phagocytic Index (PI), indicating cells with internalized particles. [24]. The Binding Index (BI) was determined by subtracting internalization counts from total counts [34], and Phagocytic Efficiency (PE) was defined as the ratio PI/AI. It is to be noted that an increase in BI could represent a decrease in efficiency. These indices are all normalized with respect to the AI for the unstimulated control cells.

### 2.3. Fluorescence Microscopy

Phagocytosis was also carried out as described above on poly-L-lysine (PLL)-coated glass slides obtained by incubation with 0.005% PLL (Sigma) for 30 min at 37 °C. Excess PLL was washed away with PBS, and the slides were left to dry. Once dry, the phagocytosis assay was carried out. The cells were then fixed with 4% paraformaldehyde for 10 min and permeabilized with TBST-Triton (10 mM Tris-HCl pH 8.0, 150 mM NaCl, 0.05% Tween 20, 0.5% Triton X-100) for 15 min. The cells were incubated with the indicated primary antibodies [rabbit polyclonal anti-sheep RBC (MP Biomedicals, Santa Ana, CA USA); rabbit IgG anti-VASP (Cell Signaling, Danvers, MA, USA); mouse IgG anti-pSer^157^-VASP (Cell Signaling) and a mouse polyclonal anti-iC3b antibody kindly donated by Dr. Santiago Rodriguez de Cordoba (CIB, CSIC, Madrid, Spain)], followed by staining with secondary antibodies (donkey anti-rabbit or donkey anti-mouse Alexa Fluor^®^ 488, 555 or 647 conjugated antibodies, (Life Technologies, Waltham, MA, USA) and TRITC-conjugated phalloidin. All antibodies were used as per the manufacturers’ instructions and in the presence of excess human gamma globulin (100 μg/mL) and 1% BSA as blocking agents. When tandem staining was required, RBCs were stained either using the rabbit polyclonal anti-sheep RBC or the mouse polyclonal anti-iC3b, depending on the compatibility with the other antibodies used. Stained cells were then observed using a LSM510 META Axiovert200 microscope (Zeiss, Oberkochen, Germany), and image analysis was performed using the ImageJ software package. Live imaging of phagocytosing cell lines was similarly performed with a LSM510 META Axiovert200 microscope (Zeiss), and the cells were maintained at 37 °C in an atmosphere of 5% CO_2_.

### 2.4. Western Blotting

To analyze protein expression, cells were harvested by centrifugation and cell lysis was carried out for 10 min at 0 °C in GST Buffer (50 mM Tris-HCl pH 7.4, 100 mM NaCl, 2 mM MgCl_2_, 10% *v*/*v* glycerol, 1% *v*/*v* NP-40) supplemented with 1 mM PMSF, 25 mM NaF, 1 mM Na_3_VO_4_ and a protease inhibitor cocktail (Sigma). Protein concentrations were determined using the RC DC™ Protein Assay kit (Bio-Rad, Hercules, CA, USA).

SDS-PAGE was carried out as described by Laemmli, loading 50 μg of total protein per lane from cell lysates. Prestained protein molecular weight standards (Bio-Rad) were used. The proteins were electrotransferred to a nitrocellulose membrane (300 mA, constant amperage, 2 h), which was then incubated overnight at 4 °C with anti-human primary antibodies rabbit IgG anti-VASP (Cell Signalling), mouse IgG anti-pSer1^57^-VASP (AbCAM, Cambridge, UK), mouse IgG anti-α-Tubulin (Sigma), IgG anti-phospho-ERK (SantaCruz Biotechnology, Dallas, TX, USA), and IgG anti-ERK, (BD Trans Lab, Franklin Lakes, NJ, USA), blocked with 5% BSA in TN-Tween (50 mM Tris-HCl pH 8.0, 150 mM NaCl, 0.05% Tween-20), and later incubated (40 min. at room temperature) with a secondary IRDye^®^ IgG anti-rabbit or anti-mouse fluorescent antibodies (Li-Cor, Lincoln, NE, USA). All antibodies were used as per the manufacturers’ instructions. The signal was then measured in a Li-Cor Odyssey imaging system and quantified using the ImageStudio software (Li-Cor).

### 2.5. Gene Silencing

A knockdown of RIAM expression using siRNA from Sigma was performed using the X-tremeGENE reagent (Roche, Basel, Switzerland). Briefly, 2 × 10^6^ cells in a 6-well plate were differentiated towards macrophage-like cells, transferred to serum-free media and incubated for 4 h with X-tremeGENE polyplexes. These consisted of 110 pmol of either target or MISSION^®^ siRNA Universal Negative Controls (Sigma) as per the manufacturer’s recommendations. After the 4 h incubation period, cell media were substituted for regular RPMI 1640 containing 10% serum. Transfection was assessed through both Western blot and functional assays.

### 2.6. Gene Knockout

Protein knockout lines were obtained using a CRISPR-CAS9 system and a double nickase strategy. Pairs of sgRNAs were designed using the Optimized CRISPR Design tool (Zhang Lab, Massachusetts Institute of Technology, Cambridge, MA, USA, 2013) [35], and the highest scoring pairs were selected. To ensure the truncated proteins were non-functional, the sgRNAs were directed towards the first common exon for all isoforms of VASP (exon 2). The corresponding pairs of sgRNAs (5’-CACCGGTAGATCTGGACGCGGCTGA-3’ and 5’-CACCGGCCAATTCCTTTCGCGTCGT-3’) and their complementary oligonucleotide chains were ordered (Sigma), annealed and ligated into a previously BbsI-digested PX458 plasmid [35]. Competent TOP10 *Escherichia coli* were transformed with the ligation mixture, and plasmids were harvested using a Wizard^®^ Plus SV Miniprep DNA purification system (Promega, Madison, WI, USA) or a Plasmid Maxi kit (Qiagen, Hilden, Germany), as per the manufacturers’ instructions.

Cell transfection was carried out using the Neon Transfection System (Thermo Fisher, Waltham, MA, USA). Briefly, cells were plated the day prior to obtain 70–90% confluency at the day of transfection. For each nucleofection, 250,000 cells and mixture of 3 μg of the two sgRNA plasmids were employed. The cells were then transfected in a 10 μL volume using a single 35 ms and 1350 V pulse, and left to recuperate for 24 h in RPMI 1640 10% FCS medium without antibiotics. The cells were then sorted according to transient EGFP fluorescence using a FACS Aria Fusion cell sorter (BD Biosciences). EGFP-positive cells were diluted and cloned into p96 wells. Protein expression was then assessed through Western blotting, and negative clones were selected.

### 2.7. VASP Overexpression

The cell lines HL-60 VASP-EGFP and HL-60 EGFP were generated through retroviral transduction using a pMSCV-EGFP-VASP plasmid kindly donated by Matthias Krause (King’s College, London, UK). To generate the pMSCV-EGFP plasmid, pMSCV-EGFP-VASP was cut and ligated to remove the VASP sequence. Correct ligation was assessed by DNA sequencing.

Retroviral particles were produced in the packaging HEK 293T cell line through transfection using 9 µg of polyethylenimine or PEI (Sigma) complexes with 3 µg of total DNA per 200,000 cells. Packaging, envelope and vector plasmid proportions were maintained as per the manufacturer’s recommendations (2:1:3; pCMV-GP/pCMV-VSV-G/vector). The supernatants containing retroviral particles were harvested and added to HL-60 cells grown to log phase and treated with polybrene (Sigma) to a final concentration of 8 μg/mL Cells were then centrifuged (2200 rpm, 90 min), grown overnight, and afterwards, cultured normally. Transfection was monitored through fluorescence microscopy. Afterwards, cells were sorted according to fluorescence at the cell sorting service at CBMSO (CSIC, Madrid, Spain) and cultured normally until a stable cell line had been established. The characterization of these cells was done via Western blotting.

### 2.8. Statistical Analysis

Figures show either representative results or mean ± standard deviation (SD) of at least 3 independent experiments (repetitions are stated in the figure legends). Significance between means was determined using the two-tailed Student’s *t*-test for independent samples (Welch’s test). Normality was assessed by using the Shapiro-Wilk test. To signal the degree of significance, asterisks are used as follows: a single asterisk denotes a significance of *p* < 0.05; a double asterisk, *p* < 0.01; and a triple asterisk, *p* < 0.005. Statistical calculations, data handling and graphing were performed with Microsoft Excel 2016 (Microsoft, Redmond, WA, USA), GraphPad Prism 6 (GraphPad Software, San Diego, CA, USA) and the VassarStats on-line resource (Vassar College, Poughkeepsie, NY, USA).

## 3. Results

### 3.1. Efficient Particle Internalization during Complement-Mediated Phagocytosis Required RIAM

We used HL-60 cell lines stably expressing RIAM shRNA (shRIAM) or control shRNA (shCtrl) [24] differentiated towards the neutrophilic linage to determine the efficiency of particle internalization during complement-dependent phagocytosis. To activate phagocytic integrins, instead of stimulating cells with bacterial moieties that induce inside-out signaling, we employed the extracellular divalent cation Mn^2+^, which binds to the metal-ion-dependent adhesive site (MIDAS), and Adjacent to MIDAS (AdMIDAS) sites in the α_M_ and α_X_ I-domains, inducing the high affinity conformation state of these integrins.

**Figure 1:**
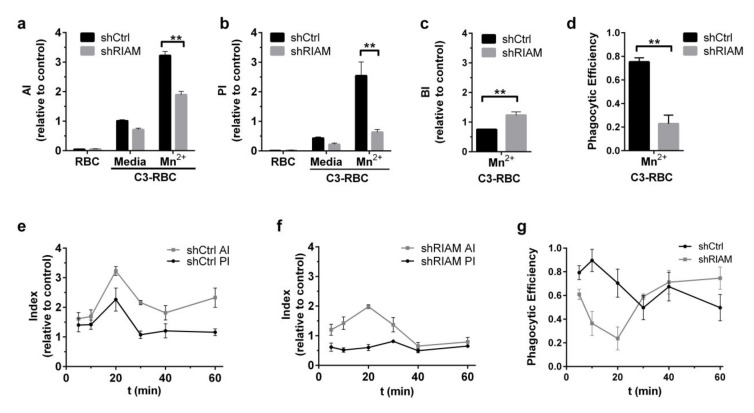
RIAM knockdown reduced complement-dependent phagocytosis after Mn^2+^ stimulation. (**a**–**d**) shCtrl and shRIAM HL-60 cells stimulated or not with 1 mM MnCl_2_ (Mn^2+^ or medium, respectively) were challenged with unopsonized (RBC) or complement-opsonized erythrocytes (C3-RBC) for 20 min. The Association (AI), Phagocytic (PI) and Binding (BI) indices and Phagocytic Efficiency were determined. These panels represent data from 10 independent experiments. (**e**–**g**) Phagocytosis assays were carried out as above, with MnCl_2_-stimulated shCtrl and shRIAM cells, which were left to phagocytose for a set amount of time, and the Association (AI), Phagocytic (PI) indices and Phagocytic Efficiency were determined. Time points represent data from five independent experiments. Data are normalized with respect to the AI of unstimulated C3-RBC challenged shCtrl cells and presented as mean ± SD. Significance (*t*-test) has been calculated with respect to controls of the shCtrl cell line; * denotes *p* < 0.05; **, *p* < 0.01.

The cells treated with MnCl_2_ or maintained in standard media were challenged with either complement-opsonized sheep red blood cells (C-RBCs) or unopsonized RBCs and allowed to phagocytose for 20 min (Figure 1a–d). Phagocytic capacity was measured using previously described indices [24]. The Association Index (AI) was determined by fluorescence from both attached and engulfed RBCs, and the Phagocytic Index (PI) represented only fluorescence from internalized particles, determined after a short hypotonic shock pretreatment. The Binding Index (BI) was calculated as the difference between the AI (total counts) and PI (internalized counts) to represent the quantity of bound non-internalized RBCs [34]. Indices are represented normalized with respect to the AI from unstimulated control cells. We also defined Phagocytic Efficiency (PE) as the relationship between the PI and AI for each cell line to determine the efficiency of particle internalization.

Mn^2+^ stimulation produced an increase in the phagocytosis of C-RBCs in both shCtrl and shRIAM cells compared with medium. However, shRIAM cells had a statistically significant reduction in the AI (41%) that was even more prominent in the PI (75%) compared to shCtrl cells (Figure 1a,b). The strong reduction in the PI was accompanied by an increment in the BI for shRIAM cells relative to that of shCtrl (Figure 1c). In accordance with the observed increase in the BI, the analysis of Phagocytic Efficiency revealed that shRIAM cells were more than three times less efficient in internalizing particles than shCtrl cells (Figure 1d). The discrepancy between the moderate reduction in association and the severely reduced efficiency suggests that RIAM plays a critical role in particle internalization that is somehow distinct from its described role in integrin activation.

To elucidate possible disturbances in phagocytic dynamics, a time-course of the AI, PI and PE was performed by allowing Mn^2+^-stimulated shCtrl and shRIAM cells to phagocytose C3-RBCs for different lengths of time (Figure 1e–g). For the shCtrl cell line, the AI reached a maximum at 20 min and experienced a fall afterward. Internalization, as determined by the PI, followed the same dynamics up to saturation at around 40 min. Phagocytic dynamics were markedly different in the RIAM-deficient cells. Whilst the AI in shRIAM cells followed similar dynamics as in control cells, albeit at a reduced rate, internalization was visibly disrupted. This was reflected by an altered phagocytic efficiency in shRIAM cells that showed a decreasing trend with minimal values at 20 min, increasing at longer time points, mainly due to markedly reduced AI values. To the contrary, control cells presented higher efficiency values with a maximum at 10 min, declining afterwards to reach the lowest values at 30 and 60 min and presenting a small increase in phagocytic efficiency at 40 min, suggestive of a recovery or recycling of the phagocytic machinery. The trend in the PE followed by shRIAM cells is suggestive of an inefficient internalization that seems to be independent of a deficiency in association, since this should result in reduced index values but not in a change in overall dynamics.

### 3.2. RIAM Knockdown Diminishes Downstream Signaling and F-Actin Enrichment at the Phagocytic Cup

**Figure 2:**
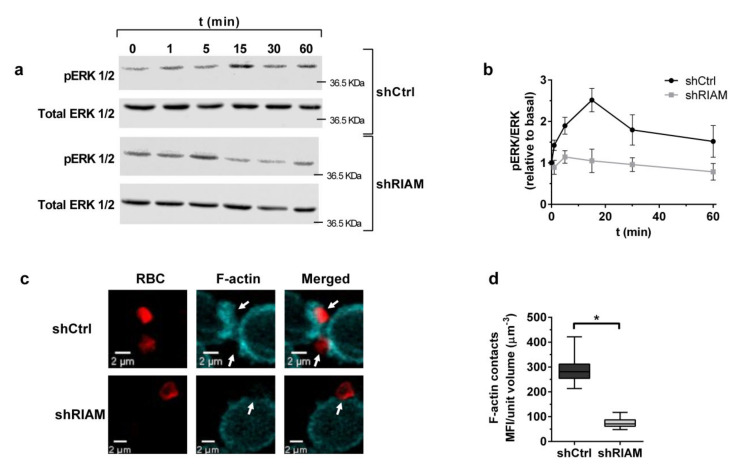
RIAM expression-determined downstream signaling and F-actin-rich phagocytic cups during complement-mediated phagocytosis. (**a**) shCtrl and shRIAM HL-60 cells stimulated or not with 1 mM MnCl_2_ were left to phagocytose for different amounts of time, the cell lysates were prepared and pERK and total ERK were analyzed by Western blotting. The figure shows representative results for five independent experiments. (**b**) Quantification of ERK activation determined as the ratio of pERK to total ERK during phagocytosis. Results are represented as relative to basal activation and are from five independent experiments. (**c**) Mn^2+^-stimulated shCtrl and shRIAM HL-60 cells were challenged with C3-opsonized RBCs, left to phagocytose for 20 min, fixed and stained for F-actin (fluorescent TRITC-phalloidin). Cells were imaged using confocal microscopy. Figure shows representative results from one confocal plane. (**d**) Analysis of quantified confocal images. Phalloidin-TRITC mean fluorescence intensity (MFI) was measured at the contact zones between cells and particles. Data are presented as mean ± SD. Significance (*t*-test) has been calculated with respect to controls of the shCtrl cell line; * denotes *p* < 0.05; **, *p* < 0.01.

As RIAM knockdown resulted in inefficient particle internalization, the downstream events in integrin signaling, such as ERK phosphorylation, were also expected to be consequently altered. To assess this, Mn^2+^-stimulated shCtrl and shRIAM cells were incubated with C3-RBC for different periods of time, and pERK and ERK were determined by Western blotting (Figure 2a,b). In shCtrl cells, ERK phosphorylation followed a pulsed response, showing a peak at 15 min and decreasing at longer times, as C3-RBCs were internalized. This contrasted with the kinetics observed in the shRIAM cells that had diminished ERK phosphorylation, reaching an early peak at 5 min and slowly decreasing over time. These results are suggestive of abnormal β_2_ integrin downstream signaling and concur with the observed reduced phagocytosis efficiency in shRIAM cells (Figure 1e–g).

F-actin enrichment is a hallmark of phagocytic cup formation and depends on β_2_ integrin downstream outside-in signaling. To determine if RIAM deficiency affects actin dynamics during phagocytic cup formation, neutrophilic shCtrl and shRIAM cells stimulated with Mn^2+^ were challenged with C3-RBCs, fixed and stained with phalloidin-TRITC. Resulting confocal microscopy images were analyzed and the fluorescence intensity of phalloidin-TRITC at RBC-cell contact sites was quantified and referred to the contact volume (Figure 2c,d). Control cells displayed F-actin-rich phagocytic cups, as well as a cortical F-actin staining pattern (Figure 2c). By contrast, shRIAM cells displayed a diffuse staining pattern with decreased F-actin at C3-RBC-cell contact sites, indicating poor actin polymerization (Figure 2c,d). These results indicate that RIAM deficiency results in the abatement of phagocytic cup F-actin content and concur with the observed reduced internalization, suggesting a functional connection between RIAM and the actin cytoskeleton.

### 3.3. Efficient Particle Internalization during Complement-Dependent Phagocytosis Required VASP Expression

**Figure 3:**
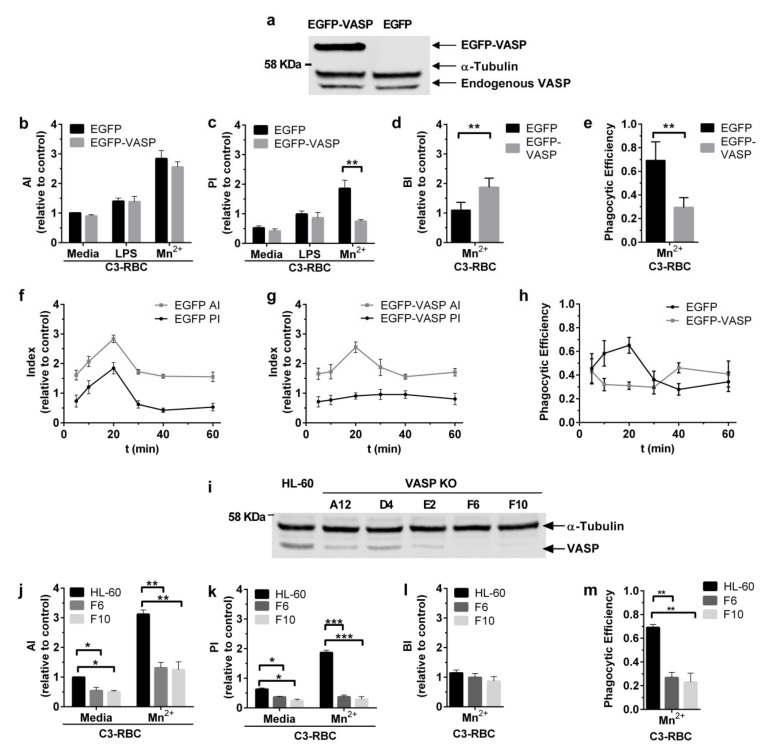
VASP overexpression and VASP knockout negatively impacted complement-mediated phagocytosis. (**a**) Newly generated HL-60 cell lines (EGFP and EGFP-VASP) were tested for VASP expression by Western blotting. (**b**–**e**) Phagocytic cells were challenged with C3-RBC after being stimulated with 1 mM MnCl_2_ or 320 nM LPS or not being stimulated (Mn^2+^, LPS or medium, respectively), and the Association (AI), Phagocytic (PI) and Binding (BI) indices and Phagocytic Efficiency were obtained. (**f**–**h**) Phagocytosis assays were carried out as above. EGFP (**f**) and EGFP-VASP (**g**) cells stimulated with MnCl_2_ were left to phagocytose for different lengths of time and the Association, Phagocytic indices and Phagocytic Efficiency were determined. All data are normalized with respect to the AI of unstimulated C3-RBC challenged EGFP cells. Each time point represents data from three independent experiments. (**i**). Newly generated HL-60 knockout monoclonal cell lines (VASP KO A12, D4, E2, F6 and F10 clones) were tested for VASP expression by Western blotting. (**j**–**m**) Phagocytic HL-60 cells and VASP KO F6 and F10 clones were challenged with C3-RBC after being stimulated with 1mM MnCl_2_ or left unstimulated (Mn^2+^ or medium, respectively), and the Association (AI), Phagocytic (PI), Binding (BI) indices and Phagocytic Efficiency were obtained. Data are normalized with respect to the AI of unstimulated C3-RBC-challenged HL-60 cells. Data are presented as mean ± SD, where the error bars denote standard deviations. Significance (*t*-test) has been calculated with respect to controls of the EGFP cell line, * denotes *p* < 0.05; **, *p* < 0.01; and ***, *p* < 0.005.

VASP has actin polymerase activity and interacts with RIAM. Therefore, VASP could be a potential candidate link to explain the connection between RIAM and F-actin. To evaluate VASP’s contribution to the phagocytic process, HL-60 cells stably expressing EGFP-VASP or only EGFP were generated (Figure 3a).

Phagocytosis assays using these cell lines stimulated with either LPS or MnCl_2_ were carried out to determine differential effects of inside-out versus outside-in induced activation. The Association (AI), Phagocytic (PI) and Binding (BI) indices were determined and are represented as relative to unstimulated EGFP cells (in standard media). Phagocytic Efficiency is also represented (Figure 3b–e). LPS-mediated inside-out stimulation did not induce any differential effects in VASP-overexpressing cells compared to their counterparts. As for outside-in stimulation with Mn^2+^, there were no remarkable differences between control EGFP and EGFP-VASP in terms of association, yet VASP overexpression significantly impaired internalization, as evidenced by the reduced PI and correspondingly increased BI and decreased PE (2.8 fold decrease). This defect in EGFP-VASP cells paralleled the blocked internalization observed for shRIAM cells (Figure 1b–d).

Since VASP is an actin polymerase, its overexpression may lead to excessive actin polymerization that might slow down and/or interfere with the phagocytic process. To test this hypothesis, the effect of VASP overexpression on phagocytic dynamics was explored. EGFP and EGFP-VASP cells were stimulated with MnCl_2_ and challenged with C3-RBC. The cells were left to phagocytose for different periods of time, and the different indices and phagocytic efficiency were determined (Figure 3f–h). Concurring with the previous results, no alterations were observed in association dynamics, as both cell lines displayed similar indices. However, the internalization dynamics were distinctly altered in EGFP-VASP cells, with the PI oscillating around a constant value of 0.88 ± 0.06, suggestive of ineffective phagocytosis. In parallel to the shRIAM phenotype, EGFP-VASP phagocytic efficiency followed a similar trend with an efficiency that oscillated around a value of 0.35, with reduced values at 10 and 20 min compared to control cells, reaching a maximum at 40 min, mainly due to a reduction in the AI.

To further elucidate the role of VASP during phagocytosis, VASP knockout clones were generated in HL-60 cells through the CRISPR/Cas9 targeting of VASP exon 2 (Figure 3i). VASP KO F6 and F10 clones were selected and stimulated with MnCl_2_ or not (medium) and the AI, PI, BI and PE were determined and represented as relative to HL-60 parental cells (Figure 3j–m). Contrasting with the overexpression phenotype, VASP KO resulted in a statistically significant reduction in the AI (Figure 3j). Particle internalization was completely abolished in VASP KO and showed no increment above basal levels after Mn^2+^ stimulation (Figure 3k). VASP KO BI levels were almost equal to those of parental cells (Figure 3l), and their phagocytic efficiencies were less than half of those of parental cells (Figure 3m), reflecting defective internalization. Taken together, these data suggest that correct VASP expression and function are critical for the effective phagocytosis of C3-opsonized targets in neutrophilic cells, highlighting the importance of this actin polymerase over other factors.

### 3.4. VASP Localized to Phagocytic Cups in a RIAM-Dependent Manner

**Figure 4:**
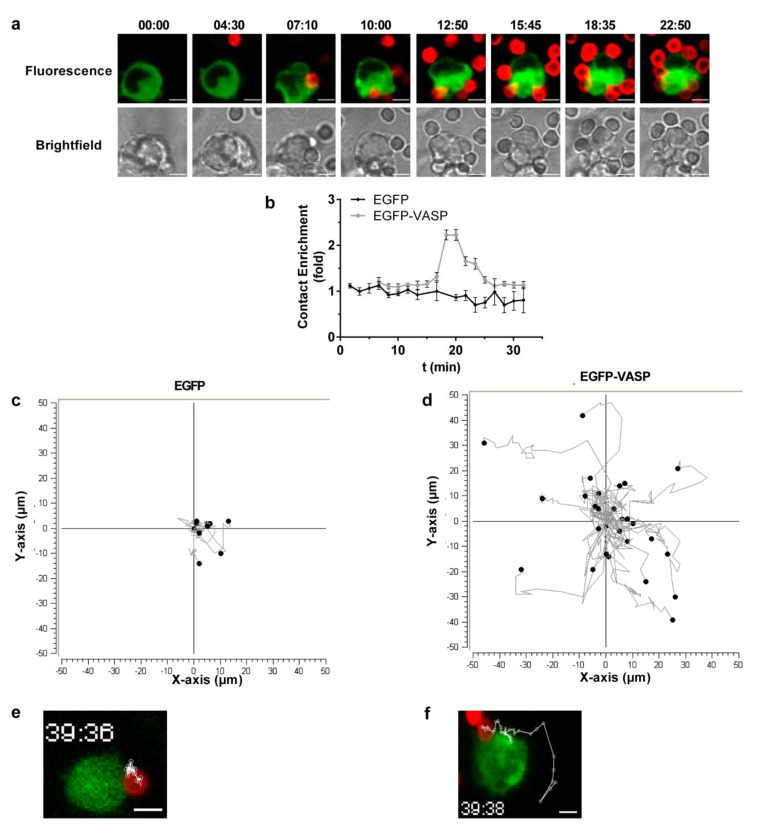
EGFP-VASP recruitment to phagocytic cups promoted overactive particle movement on cell surfaces. (**a**) Time-lapse confocal microscopy was performed on MnCl_2_-stimulated C3-RBC-challenged EGFP-VASP HL-60 cells. RBCs are shown in red and EGFP-VASP is in green. The images show representative data from a single confocal plane. Scale bars represent 5 µm. (**b**) Time-lapse images were analyzed and the contact mean fluorescence intensity was quantified and graphed. Results are represented as fold increases with respect to total cell mean fluorescence at t = 0. Represented data correspond to 50 contacts per cell line. Data are presented as mean ± SD, where the error bars denote standard deviations. Significance (*t*-test) has been calculated with respect to controls of the EGFP cell line. (**c**,**d**) RBC displacement was tracked on the x-y axis and represented for phagocytosing EGFP and EGFP-VASP cells (**c**,**d**, respectively) with *n* = 50. (**e**,**f**) Representative images of either EGFP (**e**) or EGFP-VASP (**f**) phagocytosing HL-60 cells corresponding to those analyzed in (**c**,**d**), respectively. The scale bars represent 5 µm. The images show representative data from the maximum intensity projection of all acquired confocal planes at the endpoint.

VASP has been reported to be recruited to FcγR-induced phagocytic cups [36]. To assess VASP localization during complement-dependent phagocytosis, time-lapse confocal microscopy was performed using EGFP-VASP cells stimulated with MnCl_2_ and challenged with C3-RBCs (Figure 4a). Prior to the addition of C3-RBCs, VASP appeared evenly distributed in the submembranal cortical cytoplasm. However, upon contact with C3-RBCs, VASP relocalized and became enriched at the RBC-cell contact sites. This enrichment was represented as the fold enrichment of contact mean fluorescence intensity (MFI) (Figure 4b). The contact MFI substantially increased in VASP-EGFP cells, around the 20 min time point, whilst it remained stable in the EGFP control cells. This peak coincided with the maximal particle association observed in the previous experiments.

During image recording, EGFP-VASP cells were observed to be very motile and bound erythrocytes actively moved on the cell surface during phagocytosis, whilst C3-RBCs on EGFP control cells remained much more stationary. Erythrocyte trajectories were mapped for both cell lines using the MTrackJ Image J plugin (Figure 4c–f). On control EGFP cells, C3-RBCs remained constrained and barely moved from their initial contact site, whilst on EGFP-VASP cells, bound C3-RBC underwent drastic changes in position and velocity. These long-range changes in RBC location on the surface of phagocytic cells correlated with the observed deficient internalization and suggest that VASP overexpression leads to an overactive cortical actin cytoskeleton.

**Figure 5:**
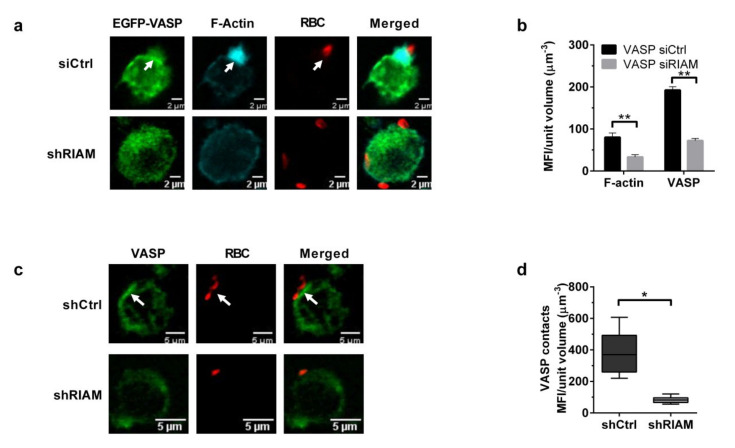
RIAM expression determined VASP membrane localization. (**a**). EGFP-VASP HL-60 cells were transfected with either control (siCtrl) or RIAM-specific siRNA (siRIAM), stimulated with 1 mM MnCl_2_ and challenged with C3-RBCs. Figure shows representative images from a single confocal plane. (**b**) Mean Fluorescence Intensity (MFI) of both EGFP-VASP and phalloidin-TRITC (F-actin content) was quantified for the contacts between cells and RBC and graphed. Data are presented as mean ± SD, where the error bars denote standard deviations. Significance (*t*-test) has been calculated with respect to the siRNA controls of the VASP cell line. Double asterisk denotes a significance of *p* < 0.01. (**c**). MnCl_2_-stimulated shRIAM and shCtrl cells were challenged with C3-opsonized-RBCs, left to phagocytose for 20 min, and then fixed and stained for endogenous VASP. RBCs were stained by using an anti-C3b antibody for this assay. Cells were imaged through confocal microscopy. The figure shows representative results from a single confocal plane. (**d**) Analysis of endogenous VASP staining intensity (MFI) at the RBC-cell contact sites quantified from the confocal images shown in (**c**). Data are presented as mean ± SD, where the error bars denote standard deviations. Significance (*t*-test) has been calculated with respect to controls of the shCtrl cell line; * denotes *p* < 0.05, and **, *p* < 0.01.

RIAM translocates from cytoplasm to the membrane during integrin activation [37]. As RIAM binds VASP, we hypothesized that this adaptor protein may be instrumental in recruiting VASP to the phagocytic cup. To establish if VASP localization was dependent on RIAM expression, EGFP-VASP cells were transfected with either a RIAM-specific or a negative control siRNA, stimulated with MnCl_2_ and challenged with C3-RBCs. EGFP-VASP and F-actin recruitment to RBC contact sites were quantified by confocal microscopy (Figure 5a,b). RIAM silencing resulted in statistically significant reductions of 58.8% in phalloidin-TRITC and 62.5% in EGFP-VASP mean fluorescence intensity at the RBC contact site. This result indicated that RIAM controlled VASP localization and may be responsible for its recruitment to phagocytic cups.

Endogenous VASP distribution was also analyzed during phagocytosis in shCtrl and shRIAM cells (Figure 5c). In control cells, VASP was located at the submembranal cortical cytoplasm, accumulating at the RBC contact site, whereas in RIAM knockdown cells, VASP presented a more diffuse pattern with a reduced MFI at the RBC contact site (Figure 5d), mirroring the pattern observed for F-actin.

**Figure 6:**
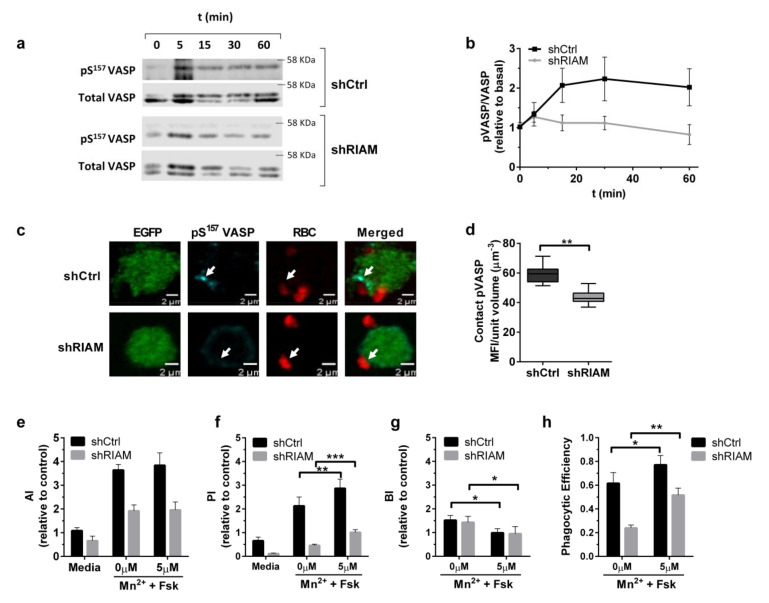
RIAM expression was required for VASP phosphorylation on Ser^157^ during complement-dependent phagocytosis. (**a**) Phagocytosis assays were carried with MnCl_2_-stimulated shCtrl and shRIAM HL-60 cells for different periods of time. Cells were lysed, and pSer^157^-VASP (pVASP) and total VASP content was determined by Western blotting. A representative experiment out of four replicates is shown. (**b**) Quantification of pSer^157^-VASP, determined as the ratio of pVASP and total VASP signal during phagocytosis at different time points in shCtrl and shRIAM cells. Results are represented as values relative to basal activation and are derived from five independent experiments. Data are presented as mean ± SD; error bars denote standard deviations. (**c**) pSer^157^-VASP localization was assessed through confocal microscopy in phagocytosing shCtrl and shRIAM cells. Arrows indicate pVASP enrichment at contacts with RBCs. The figure shows representative results from a single confocal plane. (**d**) pSer^157^-VASP mean fluorescence intensity at the RBC contact site was quantified from cells in (**c**). The figure shows results from 50 cells. (**e**–**h**) AI, PI, BI and Phagocytic Efficiency were obtained from phagocytosis assays using unstimulated (medium) or MnCl_2_-stimulated shCtrl and shRIAM cells that were either untreated (0 µM) or treated with 5 µM Forskolin (Fsk). The figure represents data from five independent experiments. Data are normalized with respect to the AI of unstimulated C3-RBC-challenged shCtrl cells. Significance (*t*-test) is calculated with respect to controls of shCtrl cells; a * denotes significance of *p* < 0.05; **, *p* < 0.01; and ***, *p* < 0.005.

VASP membrane localization is dependent on Ser^157^ phosphorylation, and in vivo integrin dependent adhesion and migration assays suggest a relationship between Ser^157^ phosphorylation and actin polymerase activity [28,31,32]. Since VASP and F-actin were reduced at C3-RBC-contact sites in shRIAM cells, the relationship between pSer^157^-VASP and RIAM expression was explored during phagocytosis. Control (shCtr) and shRIAM cells stimulated with Mn^2+^ were challenged with C3-RBC for different periods of time, and VASP and pSer^157^-VASP levels were determined by Western blotting (Figure 6a).

In control cells, VASP phosphorylation followed saturation kinetics and reached a peak at around the 30 min time point. However, in shRIAM cells, VASP showed lower phosphorylation and altered kinetics (Figure 6b). Using confocal microscopy, pSer^157^-VASP localization was assessed for shRIAM and shCtrl cells during phagocytosis (Figure 6c,d). In control cells, pSer^157^-VASP was markedly localized in proximity to the RBC contact site, whereas in shRIAM cells, pSer^157^-VASP displayed a diffuse pattern with a significant reduction in MFI at the RBC–cell contact site.

Considering that shRIAM cells have reduced pSer^157^-VASP, we proposed that an increment in VASP Ser^157^ phosphorylation might alleviate the internalization deficiency observed in shRIAM cells. To test this hypothesis, cells were treated with forskolin (Fsk), which activates PKA and increases pSer^157^-VASP levels [32]. Whilst Fsk treatment after MnCl_2_ stimulation did not provoke significant changes in the AI (Figure 6e), a statistically significant two-fold increase in the PI was observed for both cell lines (Figure 6f). This result suggested that forskolin treatment was capable of partially reverting the RIAM knockdown phenotype in terms of particle internalization as indicated by the increase in phagocytic efficiency induced by Fsk treatment (Figure 6h).

## 4. Discussion

Complement-dependent phagocytosis requires receptor activation, cytoskeletal reorganization with membrane extension and particle internalization. This process requires strict coordination between different pathways, making signaling platform molecules, such as RIAM, essential. The data presented here suggest a critical role for RIAM in the coordination of β_2_ integrin-induced actin cytoskeletal dynamics via its interaction with VASP. While the role of RIAM in CR3 activation after inside-out stimulation is already established [24], it is uncertain if RIAM is also involved in integrin outside-in signaling.

We demonstrate that RIAM knockdown reduced complement-mediated phagocytosis in cells treated with Mn^2+^, a stimulus that promotes full integrin activation and high affinity for their ligands. Under these stimulation conditions, shRIAM cells showed a 42% reduction in the association (AI) of C3-opsonized particles. This defect did not result in altered association dynamics, as shRIAM cells followed the same trend as control cells. By contrast, RIAM knockdown cells showed impaired internalization with altered dynamics. Control cells showed a pulsed response, whereas shRIAM cells exhibited a delayed and flat response. This emphasizes the importance of RIAM in particle engulfment, as a reduced expression of this adaptor molecule may slow effector recruitment, acting as a bottleneck for the process. In line with this observation, macrophages from RIAM null mice showed defective adhesion to ICAM-1 that was partially rescued by Mn^2+^ treatment. Based on these results, Klapproth et al. concluded that RIAM may be involved in outside-in signaling [25].

The defective complement-phagocytosis coincided with overall reduced pERK levels and distorted ERK phosphorylation kinetics in shRIAM cells. A reduction in this signaling pathway has been described in RIAM knockdown melanoma and breast carcinoma cell lines to cause impaired adhesion turnover that correlates with an inhibition of the MEK-ERK pathway and deficient RhoA activation [38]. The results reported herein suggest that RIAM expression determines ERK phosphorylation and its kinetics during complement-mediated phagocytosis in a manner comparable to adhesion turnover regulation for migrating cells.

Scaffolding proteins coordinate signal transmission but require specific expression levels to do so. Suboptimal concentrations of Ste5 (yeast) and of its mammalian equivalent KSR1/2, both controlling the MAPK pathway, result in delayed cell responses, due to imbalances between the concentrations of these scaffolds and their effectors [39,40,41,42]. Both the phagocytic dynamics and ERK phosphorylation kinetics of shRIAM cells are very reminiscent of these responses, further emphasizing the importance of RIAM as a signaling platform.

In addition to presenting altered ERK phosphorylation kinetics, RIAM deficient cells exhibited reduced levels of F-actin at phagocytic cups. This may explain the diminished particle internalization observed for these cells and concurs with the defects in neutrophil migration and extravasation observed for *RIAM*^−/−^ mice [25], as both processes require actin cytoskeleton remodeling. Similarly, these mice show a defective bone marrow and lymph node homing of B and T cells [43], further associating motile processes with RIAM expression.

Our results suggest that VASP plays a crucial role in particle internalization after Mn^2+^ stimulation. VASP overexpression significantly reduced the Phagocytic Index over time, suggesting altered internalization and ineffective phagocytosis. By using time-lapse fluorescence microscopy, we observed that whereas in the EGFP control cell line, bound C3-RBCs remained rather stationary, C3-RBCs bound to EGFP-VASP cells moved haphazardly (a process reminiscent of membrane ruffling), and this might explain the poor phagocytosis. In accordance with these results, exogenous VASP expression induces ruffle formation in endothelial cells [44], and Mena and VASP overexpression inhibits fibroblast cell motility [45], which the authors attribute to membrane ruffling. This phenotype is dependent on Ena/VASP protein localization. Fibroblasts in which Ena/VASP proteins are recruited to the plasma membrane (by the co-expression of a construct containing an EVH1 binding motif and a plasma membrane targeting domain) exhibit a rapid withdrawal of lamellipodial protrusions or membrane ruffles [46].

These observations concur with our data, which showed that EGFP-VASP was enriched at the phagocytic cup and was related to F-actin content, all of which were dependent on RIAM expression. The same holds true for endogenous VASP, as its localization at phagocytic cups was diminished as a result of RIAM knockdown. Our results demonstrate that VASP localization is dependent on RIAM expression, pointing to a link between these two proteins and their role in regulating complement-mediated phagocytosis.

VASP knockout cell clones presented strong defects in particle internalization, and this contrasts with prior reports stating that fibroblast migration is enhanced in the absence of all Ena/VASP proteins [45]. Another study suggests that VASP expression controls tensile strength, contractility and cytoskeletal rigidity [47]. VASP-deficient fibroblasts show thickened stress fibers and delayed but more stable adhesions. The discrepancy between our observations in complement-mediated phagocytosis and adhesion assays could be explained because phagocytosis is a more dynamic process and a delay in establishing a stable adhesion may halt the process. Furthermore, fibroblast adhesions are typically characterized by branched (Arp2/3-mediated) F-actin and lamellipodial protrusions and not by lineal filopodial extensions like those mediated by VASP or formins. These two groups of actin assembly factors may compete for a pool of profilin-bound G-actin and negatively regulate each other [48].

The fact that in our model, phagocytosis was practically abolished in VASP KO cells highlights its importance over other factors that could compensate the defect. Of special note is mDia, a formin that is recruited specifically to CR3 phagocytic cups by binding to microtubule-binding CLIP-170. At phagocytic cups, mDia1 interacts with active RhoA, which promotes local actin polymerization. In accordance, mDia1-deficient macrophages display inefficient particle internalization [49,50]. VASP and mDia are unbranched actin filament polymerases with potentially overlapping functions. However, studies in *Drosophila melanogaster* cells have revealed that both proteins drive the formation of filopodia with distinct morphology and dynamics, suggesting their roles are not redundant. Long, stable filopodia require mDia, whereas Ena (VASP homolog) promotes more dynamic elongations. Ena inhibits mDia activity, allowing cells to switch from long, persistent protrusions to dynamic mixes of lamellipodia and filopodia [51]. The differences in polymerase activity suggest that these factors may have non-overlapping functions, explaining the defects in phagocytosis observed in mDia- or VASP-deficient cells.

In addition to the previous, the actin nucleator Arp2/3 was described to participate in CR3 phagocytosis whilst being dispensable for FcγR phagocytosis in primary bone marrow-derived mouse macrophages [48]. Arp2/3 activity was shown to drive the leading edge of protrusions and phagocytic cup formation during complement phagocytosis, whereas mDia1 activity coupled advancing protrusions to the particle surface. An association between Talin and Vinculin was also demonstrated in phagocytic cups containing β_2_ integrins, with organization in structures similar to focal adhesion complexes [52].

In migrating epithelial cells, actin assembly factors act sequentially, starting with mDia1, followed by the Arp2/3-mediated formation of cortical actin, and elongation mediated by mDia2 or VASP [53]. A hierarchy is also proposed during complement-dependent phagocytosis [52], where Arp2/3 may provide the cortical actin platform for mDia activity. We propose that in complement-mediated phagocytosis, VASP, in coordination with Arp2/3 and mDia1, may work sequentially to promote balanced F-actin dynamics during phagosome formation.

VASP phosphorylation at Ser^157^ correlates with membrane localization [28,29,30,54]. We have demonstrated that RIAM knockdown produced a notable reduction in the VASP and F-actin content at the phagocytic cup, with altered VASP pSer^157^ phosphorylation kinetics and overall levels. We hypothesize that RIAM may recruit VASP to active integrins, where it may be phosphorylated. It is established that in mice cells, the non-phosphorylatable VASP (S153A) reduces cell chemotaxis [31]. Further experiments using either non-phosphorylatable or phosphomimetic mutants of VASP could corroborate if a similar mechanism operates in complement-dependent phagocytosis. Indeed, in mouse cells, RIAM co-immunoprecipitates preferentially with non-phosphorylatable VASP (S153A) [55], while Vinculin—another VASP binding partner—is unable to co-immunoprecipitate with it. To the contrary, Vinculin-VASP interactions are induced in cells treated with acetylcholine, which promotes both VASP Ser^157^ and Vinculin Tyr^1065^ phosphorylation [32]. All this suggests that the VASP phosphorylation state determines VASP’s preference for its binding partners.

The phosphorylation of VASP on Ser^157^ correlates with actin polymerization in vivo [28,31,32]. Our results from using forskolin to increase VASP phosphorylation showed an increase in phagocytosis and a partial reversion of the RIAM knockdown phenotype, suggesting increased actin polymerization. In vitro studies of recombinant VASP pSer^157^ phosphomimetics have not shown an increase in polymerase activity [30]. However, other in vitro studies using lamellipodin, a member of the MRL family of proteins involved in VASP recruitment to the lamella [56], demonstrate that this protein increases VASP processivity and therefore increases polymerization rates [57]. As RIAM is part of the MRL family, we hypothesized that a similar process occurs during complement-mediated phagocytosis, which would concur with our results.

The present work describes the role of both RIAM and VASP in complement-mediated phagocytosis. Our results demonstrate that RIAM is required for outside-in integrin signaling. We proved that RIAM acted as a relay, linking integrin activation and cytoskeletal dynamics through VASP, controlling its localization and phosphorylation status, which may in turn regulate its activity through the recruitment of different binding partners.
